# Anti-proliferative effects of raw and steamed extracts of *Panax notoginseng *and its ginsenoside constituents on human liver cancer cells

**DOI:** 10.1186/1749-8546-6-4

**Published:** 2011-01-24

**Authors:** Ding-Fung Toh, Dhavalkumar Narendrabhai Patel, Eric Chun-Yong Chan, Alvin Teo, Soek-Ying Neo, Hwee-Ling Koh

**Affiliations:** 1 Department of Pharmacy, Faculty of Science, National University of Singapore, 18 Science Drive 4, 117543, Singapore; 2 Republic Polytechnic, School of Applied Science, 9 Woodlands Ave 9, 738964, Singapore; 3 Singapore Institute for Clinical Sciences (A*STAR), 30 Medical Drive, 117609, Singapore

## Abstract

**Background:**

*Panax notoginseng *is a potential source of anticancer compounds. This study aims to investigate the effects of steaming on the chemical profile of *P. notoginseng *and the anti-proliferative effects of *P. notoginseng *on liver cancer cells.

**Methods:**

Samples of powdered raw *P. notoginseng *roots were steamed for various durations. Extracts of the raw and steamed samples were subjected to ultra-high pressure liquid chromatography/mass spectrometry (UHPLC-MS) analysis for chemical profiling. The anti-proliferative effects on three human liver cancer cells, namely SNU449, SNU182 and HepG2, were evaluated using colorimetric WST-1 assay.

**Results:**

Steaming changed chromatographic and pharmacological profiles of *P. notoginseng*, causing differences in activities such as inhibition of cancer growth. Steamed *P. notoginseng *exhibited greater anti-proliferative effects against liver cancer cells (SNU449, SNU182 and HepG2) than its raw form; steaming up to 24 hours increased bioactivities. Steaming increased the concentrations of ginsenoside Rh_2_, Rk_1_, Rk_3 _and 20S-Rg_3 _and enhanced growth inhibition of liver cancer cells.

**Conclusion:**

Steaming changes the chemical profile as well as anti-cancer biological activities of *P. notoginseng*. Steamed *P. notoginseng *contains potential compounds for the treatment of liver cancer.

## Background

Some Chinese medicinal herbs exhibit anti-tumour activities [1,2]. The raw form of *Panax notoginseng *(Burk.) F.H. Chen (*Sanqi*) is used in Chinese medicine to arrest internal and external haemorrhages, eliminate blood stasis, improve blood circulation, disperse bruises, reduce swelling and pain [3]. The steamed form, on the other hand, is used as a tonic to nourish blood by increasing the production of various blood cells to treat anaemia [3]. The roots of *P. notoginseng *which exhibited anticancer activities [4-6] were effective against colorectal [5,6], lung [7], gastric [8,9], skin [4], prostate [10] and liver [11] cancer. The ethanol extracts of *P. notoginseng *inhibited spleen tumour growth and liver metastasis *in vivo *[11]; however, the *in vitro *anti-proliferative effects of *P. notoginseng *on liver cancer have yet to be evaluated.

Ginsenosides or dammarane-type triterpenoidal saponins, the main bioactive constituents of *P. notoginseng *[12,13], are effective in preventing and treating cardiovascular and cerebrovascular diseases. These compounds are also immunoregulatory, possessing properties such as hepatoprotection and anti-carcinogenesis [14]. Ginsenoside Rd was effective against human cervical cancer [15] and 20S-25-methoxyl-dammarane-3β, 12β, 20-triol was effective against several other types of cancer [16]*in vitro*. Ginsenoside Rh_2 _inhibited the growth of human hepatoma cell SK-Hep-1 [17]. Ginsenoside Rk_1 _controlled human hepatocellular carcinoma cell HepG2 [18] proliferation. Ginsenosides Rg_3_, Rg_5_, Rk_1_, Rs_5 _and Rs_4 _are 50% more effective than cisplatin in inhibiting growth in human hepatoma cell SK-Hep-1 [19].

Processing of *P. notoginseng *(*eg *steaming) may change its composition [20-23] and alter its biological activities [6,24]. While a study showed that steaming of *P. notoginseng *increased its anticancer activities [6], the correlation between altered composition of *P. notoginseng *and growth inhibition has not been established. Moreover, the relations between compositional changes in steamed *P. notoginseng *and changes in biological activities (*eg *antiproliferation on liver cancer cells) have yet to be investigated.

This study investigates the effects of steaming on the chemical profile of raw *P. notoginseng *and the effects of steaming duration on anti-proliferative activities of *P. notoginseng *in three liver cancer cell lines.

## Methods

### Materials

Leucine-enkephalin and formic acid were purchased from Sigma-Aldrich (USA). Acetonitrile (HPLC grade) was purchased from Merck (USA). Methanol (HPLC grade) was purchased from Fisher Scientific (USA). Distilled water was prepared 'in-house' using a MilliQ system (Millipore, USA). Ginsenosides Rg_1_, Rb_1_, Rc, Rb_1_, Rd and Re were purchased from Indofine Chemical Company (USA). Dimethyl sulfoxide (DMSO) was purchased from MP Biomedicals (USA). Notoginsenoside R_1 _was purchased from the National Institute for the Control of Pharmaceutical and Biological Products (China). Rg_3 _was purchased from ChromaDex, Inc (USA). Rh_1_, Rh_2 _and Rg_2 _were purchased from Delta Information Centre for Natural Organic Compounds (China). *P. notoginseng *roots were obtained from Wenshan, Yunnan Province, China. The materials were identified to be *P. notoginseng *through morphological characteristics as well as qualitative and quantitative analyses with comparisons to the authenticated herb obtained from the National Institute for the Control of Pharmaceutical and Biological Products (Beijing, China) [20-23]. A voucher sample was kept at the Department of Pharmacy Herbarium, National University of Singapore.

Human liver cancer cell lines SNU449 (CRL-2234), SNU182 (CRL-2235) and HepG2 (HB-8065) were purchased from American Type Culture Collection (ATCC, USA) and cultured in RPMI 1640 (for SNU449 and SNU182 cells) and Dulbecco's Modified Eagle Medium DMEM (for HepG2 cells) media supplemented with 10% fetal bovine serum FBS in a humidified atmosphere of 5% CO_2 _at 37°C. Cell proliferation was evaluated using the WST-1 assay (Roche, Germany) according to the manufacturer's instructions.

### Steaming and extraction of raw P. notoginseng

Samples of the powdered raw *P. notoginseng *root were steamed at 120°C in an Hirayama Hiclave™ HV-50 autoclave (Hirayama (HMC), Japan) for 2, 6, 9, 15 or 24 hours. The powder was then vacuum dried at 80°C until constant weight and extracted with a Branson ultrasonicator (model 5510, USA) as described by Chan *et al. *[21]. Six individual extractions were performed on the raw and steamed samples to generate six replicates each of the raw and steamed extracts [21].

### Ultra-high pressure liquid chromatography (UHPLC) and mass spectrometry (MS)

Ultra-high pressure liquid chromatography (UHPLC) was performed using a Waters ACQUITY UPLC™ system (USA), equipped with a binary solvent delivery system (Waters, USA) and an auto-sampler (Waters, USA). The chromatography was performed on a 100 × 2.1 mm Waters ACQUITY C18 1.7 μm column. The mobile phase consisted of (A) 0.1% formic acid in distilled water and (B) acetonitrile containing 0.1% formic acid.

Mass spectrometry (MS) was performed using a QTOF premier™, a quadruple TOF mass spectrometer (Waters, USA). The system was tuned for optimal sensitivity and resolution using leucine-enkephalin (2 ng/μL) and syringe pump infused at 3 μl/min in negative electrospray (ES-) ionization mode. The TOF mass spectrometer was operated in the 'W' mode and tuned using the standard compound ginsenoside Rg_1_. Data were centroided during acquisition using independent reference lock-mass ions via the LockSpray™ (Waters, USA) interface to ensure mass accuracy and reproducibility. Leucine-enkephalin was used as the reference compound (2 ng/μL) at an infusion flow rate of 3 μL/min. Sodium formate was used for calibration for accurate mass. The conditions for the UHPLC analysis with MS detection were as previously reported [23].

### Method validation

A standard mixture containing ginsenosides Rb_1_, Rb_2_, Rc, Rd, Re, Rg_1_, Rg_2_, Rg_3_, Rh_1_, Rh_2 _and notoginsenoside R_1 _was prepared in 50% (v/v) methanol. A volume of 2 μl of a standard mixture was used for the validation of retention time reproducibility and mass accuracy. A blank sample consisting of 50% methanol (2 μl) was injected between analyses to validate inter-sample cross-talking effect.

### Cell proliferation analysis

Raw and steamed *P. notoginseng *extracts were dissolved in 100% DMSO. Ginsenosides were dissolved in water. Cells were seeded in 96-well plates with the optimized cell density for the three cell lines, namely SNU449 (4 × 10^3 ^cells/well), SNU182 (4 × 10^3 ^cells/well), HepG2 (1.1 × 10^4 ^cells/well). After 24 hours of incubation, various concentrations of extracts/ginsenosides were added to the wells. The final concentration of DMSO for the extracts was 0.5% (v/v). A total of 100 μl of sample was added to each well. DMSO was used as control for the extracts and water was used as control for the ginsenosides. Controls were exposed to culture medium containing 0.5% DMSO or 10% water without drugs. All experiments were performed in quadruplicates and repeated three times. Three extracts obtained separately on three independent occasions were tested in the experiments. The SNU449 cell line was treated for 48 hours while SNU182 and HepG2 cell lines were treated for 72 hours. Cell proliferation was evaluated using WST-1 assay. At the end of the drug exposure period, the medium was replaced with 100 μl of the (10% v/v) WST-1 in fresh medium in each well and incubated for one hour. Absorbance was read at 440 nm with reference at 650 nm. The effect of herbal extracts or ginsenosides on cell proliferation was calculated as percentage of cell viability measured in the presence of 0.5% (v/v) DMSO. Results are presented as a percentage of the vehicle with values being mean ± standard deviation of three individual experiments conducted in triplicates each.

### Statistical analysis

Differences in anti-proliferative activities between *P. notoginseng *samples and ginsenosides were compared against the control using one-way analysis of variance (ANOVA) and Tukey's multiple comparison post-tests (GraphPad Prism 4, USA). Spearman correlation method is used for the correlation studies of duration of steaming of *P. notoginseng *samples and its anti-proliferative activities. Results were considered statistically significant when *P *< 0.05. Inhibition concentration (IC_50_) was determined using Calcusyn software (Biosoft, UK).

## Results and Discussion

**Figure 1:**
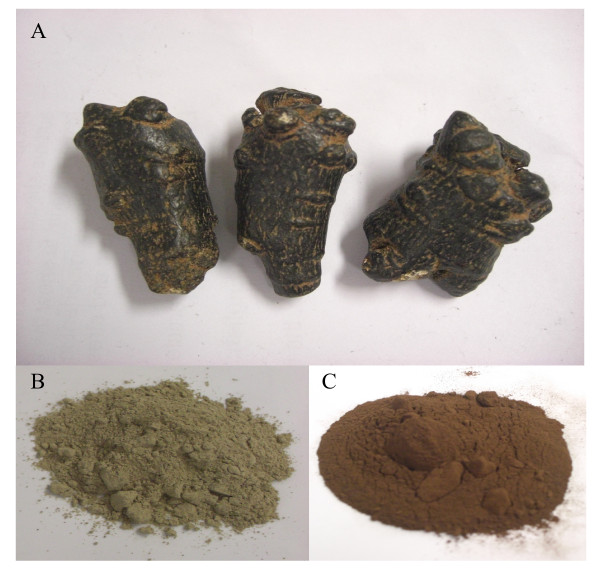
**Morphology of raw *P. notoginseng *and samples of raw *P. notoginseng *and steamed (15 hours) samples**. A: Roots of raw *P. notoginseng*. B: Raw *P. notoginseng *powder. C: Steamed (15 hours) *P. notoginseng *powder

**Figure 2:**
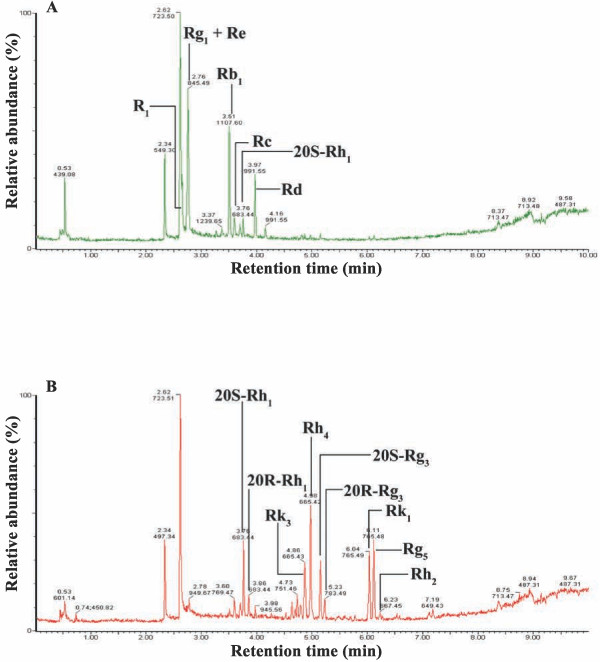
**Representative UHPLC/TOFMS chromatograms of (A) raw and (B) steamed *P. notoginseng *(15 hours)**.

Figure 1 shows the morphology of the raw *P. notoginseng*, powdered *P. notoginseng *and steamed *P. notoginseng *(15 hours). The raw and steamed *P. notoginseng *showed distinct chromatographic profiles, indicating that steaming altered the composition of ginsenosides by increasing the generation of non-polar ginsenosides which eluted later from the column (Figure 2). Chromatographic analyses of raw and steamed *P. notoginseng *were consistent with those reported by Lau *et al. *[22], who noted that the quantitative differences were correlated to the duration of steaming. Furthermore, Lau *et al *reported that the concentrations of less polar saponins such as ginsenosides 20S-Rh_1_, 20R-Rh_1_, Rk_3_, Rh_4_, 20S-Rg_3_, 20R-Rg_3_, Rk_1 _and Rg_5 _increased in steamed *P. notoginseng *[20,22]. Ginsenosides in the raw *P. notoginseng *underwent hydrolysis to form other ginsenosides upon steaming. For example, ginsenosides Rh_1_, Rh_2 _and Rg_3 _were produced from ginsenoside Rb_1 _through deglycosylation where the glycosyl moiety at C-20 was partially detached [25]. The ginsenosides Rk_1_, Rk_3 _and Rg_5 _were the examples of ginsenosides generated by the loss of water from the corresponding ginsenosides with a free hydroxyl group at C-20 [26]. One of the major saponin in raw *P. notoginseng *was ginsenoside Rg_1_. This study found that the concentration of ginsenoside Rg_1 _in the raw *P. notoginseng *was reduced from 38.9 to 1.0 mg/g upon steaming for 15 hours [27]. Taken together, these findings indicate that steaming changed the compositional profiles of the *Panax *species and altered their biological activities.

### Anti-proliferative activities of P. notoginseng extracts in liver cancer cells

The anti-proliferative effects of extracts of the raw and steamed *P. notoginseng *root were evaluated on three liver cancer cell lines. Due to the inherent different cell doubling times of the respective cell lines, the duration of treatment was optimized to 48 hours for SNU449 and SNU182, 72 hours for HepG2 respectively.

The anti-proliferative effects of *P. notoginseng *extracts on the three cell lines are in Table 1. Raw *P. notoginseng *at 0.25 mg/ml did not show any inhibition of cell proliferation in SNU182 and HepG2 whereas the growth of SNU449 cells was inhibited by about 20% (*P *= 0.018).

**Table 1:** Anti-proliferative effects (percentage viabilities) of raw and steamed *P. notoginseng *extracts on the SNU449, SNU182 and HepG2 liver cancer cells.

	SNU449		SNU182		HepG2	
*P. notoginseng*	% viability	*P *value	% viability	*P *value	% viability	*P *value
Raw	80.0 (6.5)	0.018	97.1 (6.0)	0.613	100.0 (5.2)	0.974
Steamed 2 hours	81.1 (6.6)	0.019	80.2 (5.4)	0.008	82.4 (5.4)	0.013
Steamed 6 hours	75.0 (5.0)	0.003	66.6 (3.6)	<0.001	55.9 (3.2)	<0.001
Steamed 9 hours	52.5 (6.3)	<0.001	28.7 (5.1)	<0.001	47.2 (5.2)	<0.001
Steamed 15 hours	38.8 (8.2)	<0.001	26.0 (0.9)	<0.001	26.3 (4.2)	<0.001
Steamed 24 hours	17.5 (8.0)	<0.001	21.1 (2.0)	<0.001	18.9 (5.2)	<0.001

Note: The cells were exposed to the extracts at 250 μg/ml concentration. The *P *values are with respect to the vehicle control.

In contrast, steamed *P. notoginseng *significantly inhibited the proliferation of all three liver cancer cell lines *in vitro*. The actual *P *values and percentage viabilities of the raw and steamed *P. notoginseng *extracts at 250 μg/ml are shown in Table 1. Spearman correlation study was conducted to study the correlation between the antiproliferative activities of *P. notoginseng *with duration of steaming. The increased duration of steaming on *P. notoginseng *was directly correlated with the higher inhibitory effect of the steamed extracts on cell growth. The *P *values and the correlation factor were presented in Table 2. The results of Spearman correlation study indicates that the correlation of the duration of steaming of *P. notoginseng *and its anti-proliferative effects on the three liver cancer cells were statistically significant. In addition, the steamed *P. notoginseng *demonstrated a dose-dependent growth inhibition in all three cell lines.

**Table 2:** Correlation between duration of steaming of *P. notoginseng *and anti-proliferative activities (percentage inhibition on proliferation of cells) on SNU449, SNU182 and HepG2 liver cancer cells

Cell line	***r***_***s***_	*P *value
SNU449	0.94	0.017
SNU182	1.00	0.003
HepG2	1.00	0.003

Note: Spearman correlation coefficient *r*_*s *_and *P *values are presented.

The raw *P. notoginseng *has no effects on cell growth up to a concentration of 1 mg/ml. Thus, the IC_50 _for raw *P. notoginseng *were not determined for all three cell lines. The IC_50 _value decreased as the duration of steaming increased, indicating that longer steaming led to greater anti-proliferative effects in all three cell lines (Table 3).

**Table 3:** Inhibition of proliferation of SNU449, SNU182 and HepG2 human liver cancer cells by raw and steamed *P. notoginseng *extracts

	**IC**_50_^* ^**(mg/ml)**		
*P. notoginseng*	SNU449	SNU182	HepG2
Raw	-	-	-
Steamed 2 hours	0.80	0.78	0.89
Steamed 6 hours	0.30	0.27	0.27
Steamed 9 hours	0.24	0.16	0.21
Steamed 15 hours	0.21	0.16	0.17
Steamed 24 hours	0.14	0.14	0.13

* IC_50 _= 50% inhibition concentration

Note: The cells were exposed to the extracts at 0.1, 0.25, 0.50, 0.75 and 1 mg/ml for 48 hours (SNU449 and SNU182) and 72 hours (HepG2). The IC_50 _values were determined using Calcusyn software for dose effect analysis. The IC_50 _value for raw *P. notoginseng *cannot be determined. For the steamed *P. notoginseng*, the IC_50 _decreased with the increase in the steaming duration.

### Anti-proliferative activities of ginsenosides in liver cancer cells

**Figure 3:**
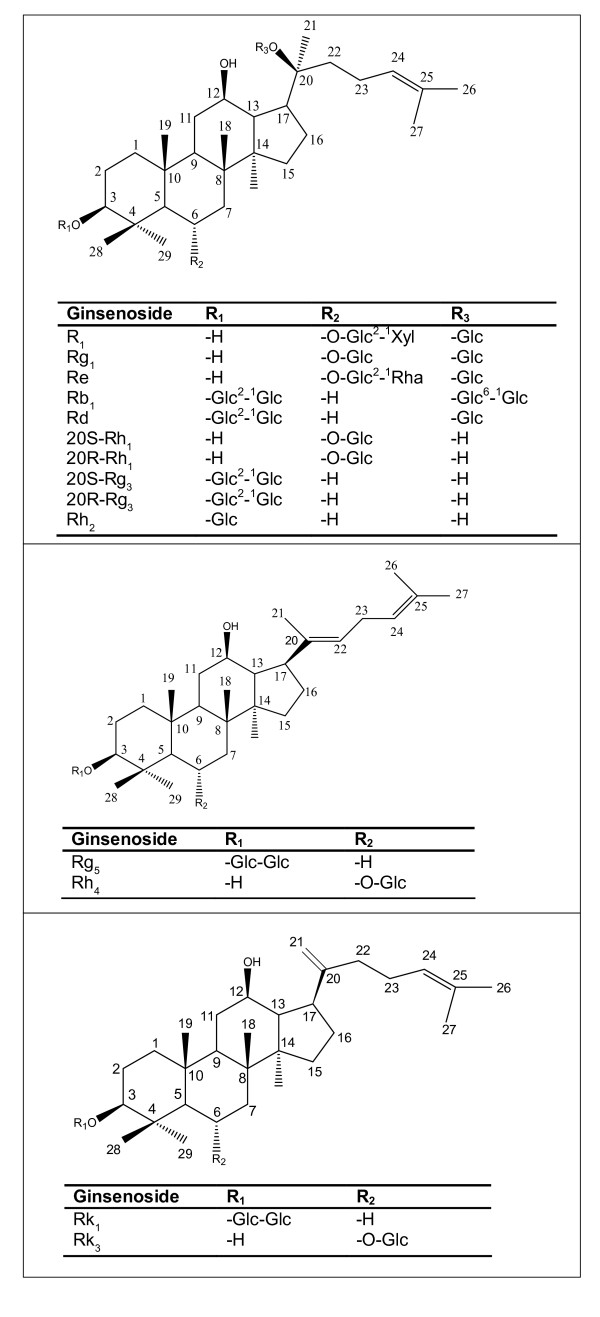
**Chemical structures of some ginsenosides and notoginsenoside in *P. notoginseng***. In raw *P. notoginseng*: notoginsenoside R_1_, ginsenoside Rg_1_, Re, Rb_1 _and Rd. In steamed *P. notoginseng*: 20S-Rh_1_, 20R-Rh_1_, 20S-Rg_3_, 20R-Rg_3_, Rh_2_, Rg_5_, Rh_4_, Rk_1 _and Rk_3_. Glc: glucose; Xyl: xylose; Rha: rhamnose

To identify the active components responsible for the anti-proliferative activities of *P. notoginseng*, we screened ginsenosides enriched in steamed *P. notoginseng*, namely 20S-Rh_1_, Rk_3_, Rk_1_, 20S-Rg_3_, Rh_2 _and 20R-Rh_1_. Ginsenosides Rg_1_, Rb_1_, Rd, Re and notoginsenoside R_1 _which predominate in raw *P. notoginseng *were also assessed for comparison. The structures of these ginsenosides and notoginsenoside are shown in Figure 3.

**Figure 4:**
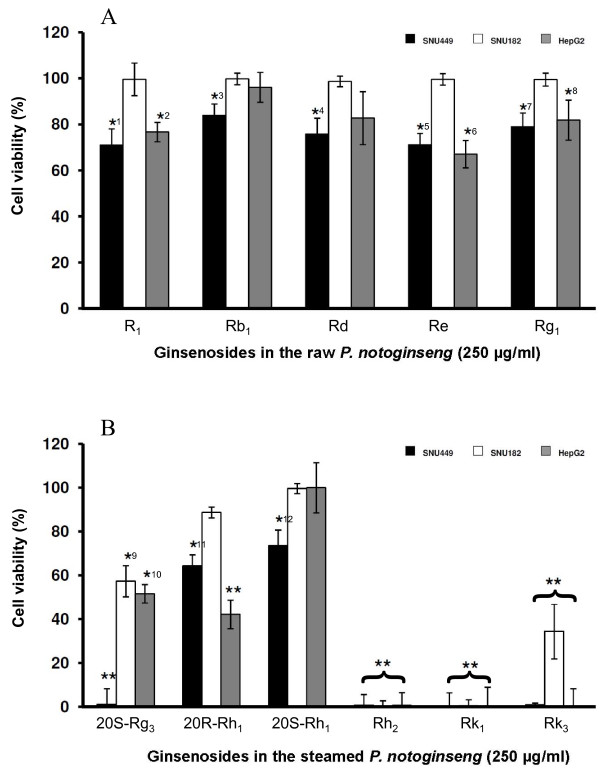
**Anti-proliferative effects of the ginsenosides from *P. notoginseng***. *In vitro *anti-proliferative effects of ginsenosides in the raw (A) and steamed (B) *Panax notoginseng *extracts in SNU449 (black square), SNU182 (white square) and HepG2 (grey square) human liver cancer cells. The cells were exposed to these ginsenosides at 250 μg/ml for 48 hours (SNU449 and SNU182) or 72 hours (HepG2) and assayed by WST-1. Plot shows the average percentage cell viability ± standard deviation as compared to vehicle control (100 ± 4.5% viability) of three independent experiments conducted in triplicates each. Statistical significance was considered when *P *< 0.05 (*) and *P *< 0.001 (**). (A) Ginsenosides Rg_1_, Rb_1_, Re, Rd and notoginsenoside R_1 _in the raw *P. notoginseng *were screened. Most of the ginsenosides in the raw *P. notoginseng *showed significant anti-proliferative activities against SNU449 and HepG2 but not SNU182. *1: *P *= 0.007; *2: *P *= 0.012; *3: *P *= 0.016; *4: *P *= 0.010; *5: *P *= 0.002; *6: *P *= 0.002; *7: *P *= 0.010; *8: *P *= 0.048. (B) Ginsenosides Rk_3_, 20R-Rh_1_, Rh_2_, 20S-Rg_3_, Rk_1_, 20S-Rh_1 _in the steamed *P. notoginseng *were screened. Most of these ginsenosides showed significant anti-proliferative effects on SNU449, SNU182 and HepG2 with ginsenosides Rh_2_, Rk_1_, Rk_3 _and 20S-Rg_3 _being the more active ones. Overall, ginsenosides in the steamed *P. notoginseng *showed greater anti-proliferative activities than ginsenosides in the raw *P. notoginseng*. *9: *P *= 0.017; *10: *P *= 0.002; *11: *P *= 0.003; *12: *P *= 0.005

The ginsenosides in the raw *P. notoginseng*, namely Rg_1_, Rb_1_, Re, Rd and notoginsenoside R_1_, exerted different growth responses in all three cell lines (Figure 4A). At 0.25 mg/ml, all of them reduced cell growth by 20-30% in SNU449 cell (Figure 4A). In SNU182 cells, cell viability was not significantly affected by any of the ginsenosides. Ginsenoside Rg_1_, Re and notoginsenoside R_1 _exerted significant anti-proliferative effects, resulting in lowered cell viabilities of 65-85% in the HepG2 cell line.

Ginsenosides Rk_3_, Rh_2_, 20S-Rg_3 _and Rk_1 _enriched in steamed *P. notoginseng *all significantly inhibited liver cancer cell growth (*P *< 0.001) at 0.25 mg/ml (Figure 4B). Ginsenoside Rk_3 _reduced cell viabilities of HepG2, SNU449 and SNU182 to 0.04 ± 0.3% (*P *< 0.001), 1.0 ± 1.2% (*P *< 0.001) and 34.4 ± 9.3% (*P *< 0.001) respectively. Ginsenosides Rh_2 _and Rk_1 _were the most potent among the four ginsenosides examined as exposure to 0.25 mg/ml of Rh_2 _resulted in minimal cell viability in all three liver cancer cells (0.4-1.4%; Figure 4B). Ginsenoside 20S-Rg_3 _effectively inhibited cell growth of SNU449 (*P *< 0.001) but was comparatively less effective on SNU182 and HepG2 cells whereas ginsenoside Rk_3 _inhibited the growth of SNU449 (*P *< 0.001) and HepG2 (*P *< 0.001), and to a lesser extent SNU182 cells (*P *< 0.001). Lastly, ginsenoside 20R-Rh_1 _significantly inhibited the growth of SNU449 (*P *= 0.003) and HepG2 (*P *< 0.001) while ginsenoside 20S-Rh1 inhibited the growth of SNU449 cells only (*P *= 0.005).

The differences in the anti-proliferative activities among the ginsenosides in the raw and steamed *P. notoginseng *are consistent with the findings that extracts of raw and steamed *P. notoginseng *show differential anti-proliferative activity on liver cancer cell lines.

The present study reports for the first time the *in vitro *anti-proliferative activities of ginsenoside Rk_3_.

### Dose-response of selected ginsenosides on the SNU449 cell line

The dose-response of four constituent ginsenosides, namely Rh_2_, Rk_1_, Rk_3 _and 20S-Rg_3_, were further assessed for efficacy for inhibiting growth in the SNU449 cell line. The IC_50 _values for ginsenosides Rh_2_. Rk_1_, Rk_3 _and 20S-Rg_3 _are listed in Table 4. As expected, ginsenoside Rh_2 _was the most potent, followed by Rk_1_, Rk_3 _and 20S-Rg_3_. The IC_50 _values of ginsenosides Rh_2_, Rk_1 _and Rk_3 _were lower than the IC_50 _of the most potent steamed *P. notoginseng *extract which is the 24 hour steamed sample.

**Table 4:** Inhibitory concentration of different ginsenosides on proliferation of SNU449 Human Liver Cancer Cells

	**IC**_50_*	
Ginsenoside	mg/ml	μM
Rh_2_	0.04	55.45
Rk_1_	0.08	100.00
Rk_3_	0.12	186.96
20S-Rg_3_	0.16	199.15

* IC_50_= 50% inhibition concentration (calculated using Calcusyn software)

Note: The cells were exposed to the ginsenosides at 0.1, 1, 5, 10, 50, 100, 200 and 250 mg/ml for 48 hours. Ginsenoside Rh_2 _showed the lowest IC_50 _value, followed by Rk_1_, Rk_3 _and 20S-Rg_3_.

### Steaming selectively enriched growth-inhibiting ginsenosides

**Figure 5:**
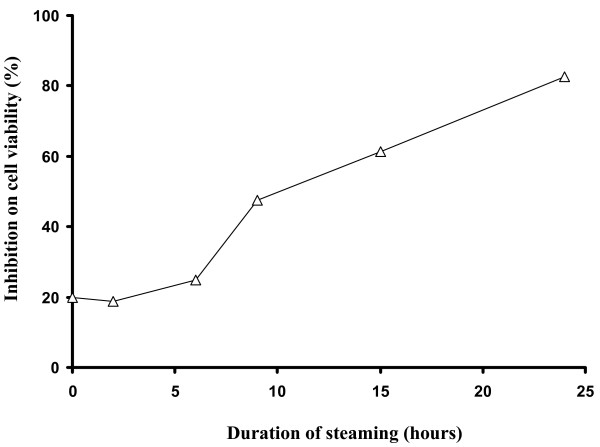
**Percentage inhibition on cell proliferation of *P. notoginseng *with different duration of steaming**. The antiproliferative activity increases with increasing duration of steaming of *P. notoginseng *sample.

**Figure 6:**
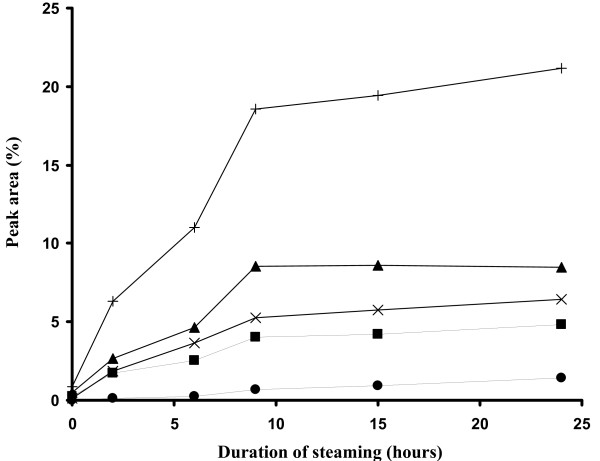
**The changes of the total peak area (plus sign) and individual peak area of ginsenoside Rk**_**3 **_**(triangle), Rk**_**1 **_**(cross), 20S-Rg**_**3 **_**(square) and Rh**_**2 **_**(circle) in *P. notoginseng *extracts with the duration of steaming**.

The anti-proliferative effects of the steamed *P. notoginseng *were positively correlated with the duration of steaming (Figure 5). The longer the steaming duration is, the greater the anti-proliferative effects. Increasing the steaming duration resulted in the formation of more ginsenosides Rk_3_, Rk_1_, 20S-Rg_3 _and Rh_2 _as indicated by the increased peak area of the four ginsenosides (Figure 6). The duration of steaming *P. notoginseng *powder correlated with increasingly enriched ginsenoside Rk_3_, Rk_1_, 20S-Rg_3 _and Rh_2 _in the extracts. Cellular exposure to these extracts resulted in a dose-dependent reciprocal inhibition of cell proliferation and cell viability. These indicate that active components were increasingly generated upon steaming, thereby increasing the anti-proliferative activities. The varying proportions of the active components in the *P. notoginseng *extracts resulted in differences in their anti-proliferative activities.

The present study demonstrated distinctive chemical profiles between the raw and steamed *P. notoginseng*. The steamed herb was significantly more effective in inhibiting the growth of liver cancer cells. The anti-proliferative activities of *P. notoginseng *increased with progressive steaming up to 24 hours as this process enriched the bioactive components such as ginsenosides Rh_2_, Rk_1_, Rk_3 _and 20S-Rg_3_.

Sun *et al. *[6] also reported that steaming the root of *P. notoginseng *affected its chemical composition and anticancer and anti-proliferative activities in SW-480 human colorectal cancer cells. However, the durations of steaming in their study were shorter (120°C for 1, 2, 4 and 6 hours) and the cancer cell line was different from what was used in our study. Furthermore they only studied three ginsenosides (Rb_1_, Rg_1 _and Rg_3_), of which only 20S-Rg3 showed significant anti-proliferative effects against SW-480. In the present study, the durations of steaming were longer (120°C for 2, 6, 9, 15 and 24 hours) and three human liver cancer cell lines (HepG2, SNU449 and SNU182) were used. In addition, more saponins were investigated for their anti-proliferative activities. In particular, Rk_3_, Rh_2_, Rk_1 _and 20S-Rg_3 _were the most anti-proliferative and ginsenoside Rk_3 _was reported for the first time to possess anti-proliferative activities.

It would be of future interest to investigate the *in vivo *anti-proliferative effects of raw and steamed *P. notoginseng *and the saponins enriched by the steaming process.

## Conclusion

Steaming changes the chemical profile as well as anti-proliferative biological activities of *P. notoginseng*. Steamed *P. notoginseng *contains potential compounds for the treatment of liver cancer.

## Abbreviations

The abbreviations used include the following: (ANOVA): analysis of variance; (DMSO): dimethyl sulfoxide; (MS): mass spectrometry; (UHPLC): ultra-high pressure liquid chromatography; (UHPLC-TOFMS): ultra-high pressure liquid chromatography - time-of-flight mass spectrometry and 50% inhibitory concentration (IC_50_).

## Competing interests

The authors declare that they have no competing interests.

## Authors' contributions

DFT designed the study, conducted the experiments, performed the statistical analyses and drafted the manuscript. PDN isolated and purified some of the ginsenosides screened in the study. ECYC and AT revised the manuscript. SYN and HLK also designed the study and revised the manuscript. All authors read and approved the final version of the manuscript.
